# Application of community-based and integrated strategy to reduce malaria disease burden in southern Tanzania: the study protocol of China-UK-Tanzania pilot project on malaria control

**DOI:** 10.1186/s40249-018-0507-3

**Published:** 2019-01-08

**Authors:** Duoquan Wang, Prosper Chaki, Yeromin Mlacha, Tegemeo Gavana, Mihayo Gabriel Michael, Rashid Khatibu, Jun Feng, Zheng-Bin Zhou, Kang-Ming Lin, Shang Xia, He Yan, Deus Ishengoma, Susan Rumisha, Sigbert Mkude, Renata Mandike, Frank Chacky, Charles Dismasi, Salim Abdulla, Honorati Masanja, Ning Xiao, Xiao-Nong Zhou

**Affiliations:** 1 National Institute of Parasitic Diseases, Chinese Center for Disease Control and Prevention, Shanghai, 200025 People’s Republic of China; 2 Ifakara Health Institute, Dar es Salaam, Dar es Salaam, United Republic of Tanzania; 3 Guangxi Center for Disease Control and Prevention, Naning, People’s Republic of China; 4 National Institute for Medical Research, Dar es Salaam, United Republic of Tanzania; 5 National Malaria Control Programme, Ministry of Health, Community Development, Gender, Elderly and Children, Dar es Salaam, United Republic of Tanzania

**Keywords:** Malaria, Community-based, Chinese experiences, WHO-T3 initiative, Tanzania, Pilot, Protocol

## Abstract

**Background:**

During the past six decades, remarkable success on malaria control has been made in China. The major experience could be shared with other malaria endemic countries including Tanzania with high malaria burden. Especially, China’s 1–3-7 model for malaria elimination is one of the most important refined experiences from many years’ efforts and key innovation measures for malaria elimination in China.

**Methods:**

The China-UK-Tanzania pilot project on malaria control was implemented from April, 2015 to June, 2018, which was an operational research with two communities receiving the proposed interventions and two comparable communities serving as control sites. The World Health Organization “Test, Treat, Track” (WHO-T3) Initiative, which calls for every suspected case to receive a diagnostic test, every confirmed case to be treated, and for the disease to be tracked, was integrated with Chinese experiences on malaria control and elimination for exploration of a proper model tailored to the local settings. Application of China’s 1–3-7 model integrating with WHO-T3 initiative and local resources aiming at reducing the burden of malaria in terms of morbidity and mortality by 30% in the intervention communities in comparison with that at the baseline survey.

**Discussion:**

The China-UK-Tanzania pilot project on malaria control was that at China's first pilot project on malaria control in Africa, exploring the feasibility of Chinese experiences by China-Africa collaboration, which is expected that the strategies and approaches used in this project could be potential for scaling up in Tanzania and African countries, and contribute to the acceleration of malaria control and elimination in Africa.

**Electronic supplementary material:**

The online version of this article (10.1186/s40249-018-0507-3) contains supplementary material, which is available to authorized users.

## Multilingual abstracts

Please see Additional file 1 for translations of the abstract into the six official working languages of the United Nations.

## Background

China has made a remarkable progress towards elimination of malaria, with six decades’ efforts including governmental leadership, policy formulation, strategies tailored to local settings, capacity building and intersectoral cooperation which have played an important role in the national malaria control and elimination programme. Especially, China’s 1–3-7 model is one of the most important refined experiences from many years work and a key innovation measure which has hugely contributed to successful national malaria control programme in China [1]. From tens of millions of cases in the 1950s, China has recorded zero cases of locally acquired infection in 2017 [2]. Meanwhile, inadequate progress has been made in most of sub-Saharan Africa where malaria remains one of the major health problems. According to the World Malaria Report (WMP) 2017, 216 million cases of malaria and 445 000 deaths which were reported globally, 90% occurred in sub-Sahara Africa [3]. To eliminate malaria in Africa, more efforts and novel approaches are called for. It is presumed that Chinese model and strategies on surveillance response systems could be adopted to control malaria in the epidemic countries of Africa.

Tanzania is one of countries of sub-Saharan Africa which is located in the central eastern part of Africa. Tanzania's health system is a central-district government structure. The ministry of Health, Community Development, Gender, Elderly and Children (MoHCDEC) is responsible for policy making and guideline development to facilitate policy enforcement. The Council Health Management Team (CHMT) is responsible for delivering health services through health structures including dispensaries, health centers and hospitals. A dispensary serves a population of 6000–10 000 people, a health center 50 000–80 000 and a district hospital 250 000. The regional referral hospital serves as a referral center to 4–8 district hospitals and the four zonal referral hospitals serve several regional referral hospitals [4, 5].

In Tanzania as it is the case with the rest of sub-Saharan Africa, malaria is a major cause of morbidity and mortality especially among children less than five years and pregnant women [6]. Malaria situations are very diverse as a result of local heterogeneities in the determinants of malaria transmission dynamics and the great variety of their local combinations. Both global and local reports demonstrated an over 50% reduction in predicted mean population-adjusted parasite prevalence among children aged 2–10 years (PA*Pf*PR_2–10_) across Tanzania between 2000 and 2012 [7]. The proportion of Tanzania’s population in intense transmission areas (PA*Pf*PR_2–10_ ≥ 50%) has declined from 11.6% to 2.3% by 2012 [7, 8]. However, the dramatic decline in malaria transmission intensity has not been witnessed uniformly everywhere; there are areas that have been experiencing a much slower epidemiological transition mostly located in the southern and north-western regions of Tanzania [9].

Elimination of malaria remains a long-term goal and mission in Tanzania. The mission envision all Tanzania have access to quality, effective, safe and affordable malaria interventions through timely, sustainable collaborative efforts among partners and stakeholders at all levels. Tanzania’s malaria control strategic plan 2014–2020 targeted a national average reduction of malaria prevalence from 10% in 2014 to 5% in 2016 and further to less than 1% in 2020 [7]. However, there are many challenges on reaching and sustaining the universal coverage policy by effective curative and preventive services. Like other resource limited countries of Africa, the heath system in Tanzania are still weak. Effective treatment is undermined by the capacity of the health system to deliver appropriate care. Stock-out reports of essential medical products for malaria and other diseases are prominent. Previous studies have reported healthcare workers' distrust on malaria test results especially when the results are negative. Correct dosing of recommended antimalarial is reported to be sub-optimal in some cases [10–12]. Most malaria cases are clinically diagnosed and treated without confirmation by either rapid diagnostic test (RDT) or microscopy, due to factors that health informarion and disease surveillance and response systems are still poor. All these systems deficiencies are not only affecting the quality of malaria case management, but also hindering the progress in malaria control, especially in achieving the proposed “Test, Treat, Track” (WHO-T3) Initiative [13]. On the other hand, ensuring the proper diagnosis, treatment and monitoring of malaria cannot be easily done by the coutry’s own systems and resources.

Leveraging on existing partnerships between Tanzania and China, this project explored the applicability and effectiveness of Chinese models and strategies, combined with local resources and WHO-T3 Initiative as a community-based approach, collaboratively established and implemented in Tanzania. The goal of the project was to explore an appropriate model and mechanism on how Chinese models and strategies could be used effectively to reduce the disease burden of malaria based on the existing local system, which could then be scaled up and applied in other similar settings of Africa. This paper describes the methodology.

## Methods

### Study areas

**Fig. 1:**
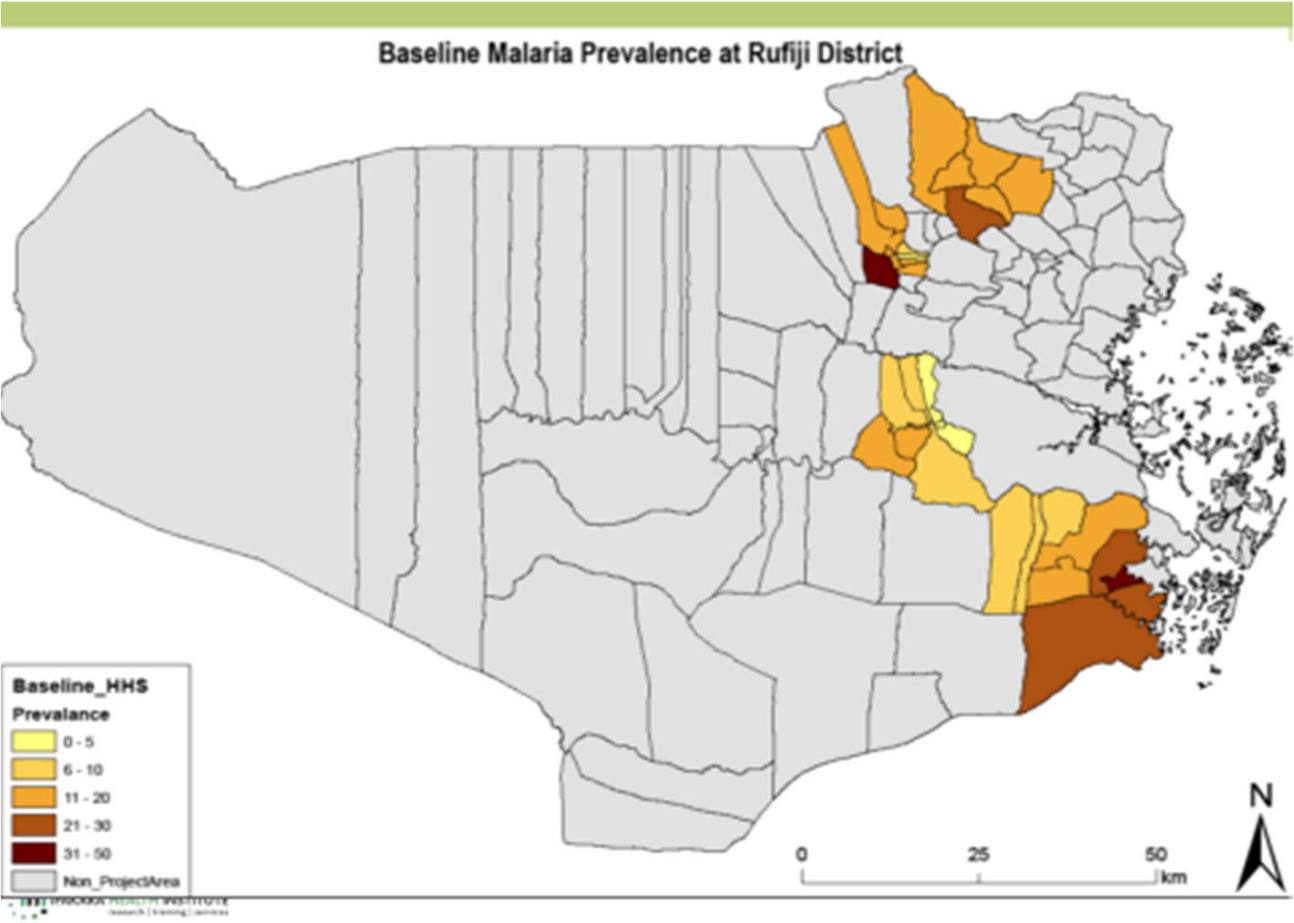
Map of malaria prevalence in the study areas

Rufiji is one of the 6 districts of the Pwani Region of Tanzania, extending from 7.470 to 8.030 south latitude and 38.620 to 39.170 east longitude, located in 178 km south of Dar-es-Salaam including six divisions with 19 wards divided into 94 registered villages with 385 hamlets. Rufiji District has an overall mean altitude of less than 500 m from sea level. Its naturally occurring vegetation is mainly formed of tropical forests and grassland. The climate in the district is hot throughout the year and there are two rainy seasons, namely, short rains (October to December) and long rains (February to May). The average annual precipitation in the district is between 800 to 1000 mm. A prominent feature in the district is the Rufiji River with its large flood plain and the delta area, which is the most extensive in the country. The public health system comprises a network of about 70 dispensaries, five health centers, and two hospitals offering a varying quality of care (see Fig. 1).

### Study design

This project aims to improve the malaria cases diagnosis, reporting, treatment and tracking through adapting the China’s 1–3-7 model. In the pilot areas, the following interventions has been take: (i) enhancing case detecting capability by increasing parasitological examination rate of all suspected malaria cases at community and health facility level; (ii) improving case management capacity by training local health workers and mobilizing communities; (iii) establishing a surveillance network for malaria parasite and vectors by an electronic reporting system, to strengthen information sharing and guiding the interventions; assessing the efficacy and cost-effectiveness of the WHO-T3 initiative through multi-factor analysis on the paired data obtained, and a final evaluation by external expert team. A prospective, baseline-endline study design was used to assess the impact of the the community-based integrated strategy in the pilot areas. This was an intervention study or operational research in two separate representative pilot communities receiving the proposed interventions and two comparable communities serving as control sites.

The study ran for three years, from April 2015 to June 2018, capturing the inter-annual and seasonal variations in transmission intensity and cumulative effects on vector populations and disease burden. All households in the clusters were recruited into the study upon written informed consent from the household head to enable all residents in the study communities to benefit from this intervention. Study staff screened clinical malaria in the communities of the pilot areas for malaria parasites by using malaria AgPf/Pan RDT as per Tanzania guidelines [7]. All participants were advised to seek treatment at a dedicated health facility for any febrile illness between scheduled visits and treatment at the dedicated study health facility and community-level mobile test stations followed the national guidelines for malaria treatment. Any participant in the home diagnosed with malaria or anaemia during screening was treated accordingly.

### Selection of participants

All people living in the selected household were included in the survey, excluding children under six months. Systematic screening of all the inhabitants of each selected household who were present at the time of the survey and consent to participate, was carried out to determine their malaria infection status.

### Sample size and power calculation

Sample size was estimated on a baseline malaria incidence rate of 0.12 cases/person/year from health facility's data. Assuming the intervention package would have an impact of 30% against malaria incidence, it was estimated to observe this effect at 5% significant level, with 80% certainty and to adjust for spatial-temporal heterogeneity in transmission, at least 18 clusters of 100 participants were required from the intervention areas.

### Data collection

Data were collected and recorded according to Social Insurance Number code and Holder Identification Number. Demographic data (age, place of birth, and geographic status), household census data, travel habits, past medical history, drug allergies and drug history, vital signs, symptoms, physical exam and lab results of study subjects (malaria infection) were collected during enrolment. All of adverse event (AE) and serious adverse events (SAE) experienced by study subjects, whether or not related to the intervention, were captured in AE and SAE form.

### Data capture

Standardized data forms or tablets were used to collect data with paper back up available in case of tablet failure. The principal investigator (PI) was responsible for the accuracy of all data entered on the forms. Any changes made on the forms were presented to the study sponsor PI and the Institutional Review Board (IRB). Training sessions were conducted with study personnel to instruct on proper input of data to ensure data integrity in line with Good Clinical Practice. In addition, the project leader conducted regular spot checks to ensure validity.

### Data storage

Research records for all study subjects including history and physical findings, laboratory data, and results of consultations were maintained in a secure facility where they stored for 10 years or until notified by grantee as per Tanzania ethical guidelines. The grantee was notified in writing and acknowledgment prior to destruction or relocation of research records. All raw and cleaned data forms were archived in the project office at Ikwiriri Rufiji in a dedicated filing cabinet and all data files were retained on the Ifakara Health Institute (IHI) central data server in accordance with IHI guidelines.

### Data validation

All paper data was converted to digital form by double-entry method to facilitate cross-referencing and validation. Entries were compared and the discrepancies found between the two databases were resolved by checking source documents. The electronic entered data, such as tablets, were entered only once. Data were checked for quality before incorporation into central database. This included standardizing dates and time, pieces of Boolean data, and used reference tables where applicable. Verified and original raw data were uploaded to the file repository to provide a documented history of data.

### Data analysis

This study was sequential in approach and founded on event-driven analyses following a predefined analysis plan conducted by independent statisticians. Interim analyses were divided into “formal” and “informal” analyses. Formal analyses used for rigorous rejection boundaries for the primary endpoint (first time infection), while all other interim analysis were considered as informal one and no hypothesis testing was performed. Descriptive statistics, including mean, SD, median and range, and interquartile were provided for continuous endpoints, and frequency and percentage were provided for categorical endpoints.

## Discussion

This is the first time that China launch a collaborative pilot project on malaria control in Africa, in order to test the feasibility of applying Chinese experiences there in the form of China-Africa health collaboration. The biggest challenge of the project is to understand how to conduct control activities in local health system and integrate this pilot project with the local resources. The experiences and lessons learnt from this pilot project will facilitate a local-tailored model of China aid, which could be scaled up to other Tanzania regions or other African countries.

China had improved its people's health with limited financial and human resources during the past 60 years since the founding of the People’s Republic of China in 1949. These achievements were due to the development and implementation of appropriate health policies emphasizing primary healthcare, infectious disease control and strengthening grass-root healthcare networks, as well as the development and implementation of other astute public policies, such as education, nutrition, water and sanitation as well as poverty reduction. While China’s health development has been promoted significantly, global health challenges have also been evolving rapidly. Recently, Chinese government agencies and international philanthropic organizations/communities have recognized that the best practices and lessons learnt from China’s health development over the past six decades could be relevant and very useful in supporting the achievement of health-related Millennium Development Goals and post-2015 Sustainable Development Goals in the low and middle income countries. Against this background, Ministry of Commerce of the People’s Republic of China, in collaboration with Department for International Development (DFID) of United Kingdom Government, has launched “Global Health Support Programme (GHSP)”, which is a China-UK partnership contributing to improved global health policy and outcomes. This pilot programme aimed at promoting China’s experiences, and exploring the new mode of tripartite cooperation through conducting malaria control pilot project in Tanzania, financially supported by DFID.

China-Africa health cooperation is an important part between African countries and China with a symbol of China-Africa Cooperation Forum established in 2000. Till now, China-Africa cooperation has reached unprecedented depth and breadth in health field. In August 2013, the Ministerial Forum of the China-Africa Health Development was held in Beijing, and the forum issued the “Beijing Declaration of the Ministerial Forum of China-Africa Health Development”(hereinafter referred to as the “Beijing Declaration”). According to the “Beijing Declaration”, African countries decided to take a series of measures to promote the development to deepen China-Africa cooperation in health, specifically including joint development of health human resources, and promote China-Africa cooperation in vocational and technical training; support African national health policies and programs; support African medicine business cooperation, encourage technology transfer and strengthen global health coordination and cooperation, including support China-Africa collaborative projects on disease control including malaria, schistosomiasis and HIV/AIDS [14].

China has accumulated lessons from the national malaria control programmes in the past six decades. Currently, Africa is expanding its scope and scale of international health cooperation, so that China is ready to share the experiences gained in malaria control and elimination stages, and expect African countries like Tanzania to be able to effectively reduce the burden of malaria disease with local health systems. The future of malaria control and elimination in mainland Tanzania will depend on a carefully defined set of evidence-based objectives, based on past experiences, present and future predictions of epidemiological conditions to target the future populations for intervention packages. The performance of this proposed project was optimized through establishing an integrated surveillance system linking three layers of information, such as incidence, epidemiological and entomological data. In addition, the project was expected to optimize the health information system through integrating the collection, collation and reporting of individual level information, which was the key to identifying and carefully targeting areas with much higher risks. This addressed gaps has been highlighted in the current strategic plan issued by the National Malaria Control Programme (NMCP) of Tanzania on strenthening collaboration with its partners, developed in 2013 to cover the period 2014–2020 [15].

In combination with the potential resources under local situation as well as the malaria epidemic status in the pilot sites in Tanzania, this project is expected to improve the surveillance systems and reduce the malaria disease budern in local settings. For improving the malaria surveillance systems, it will improve the malaria cases diagnosis, reporting, treatment and tracking using the China’s 1–3-7 model with slight modification to fit within the local existing system. For effectively reducing local malaria disease burden, application of WHO-T3 iniatative integrating with the Chinese experiences on malaria control and elimination will be explored in the pilot areas. These pilot experiences will provide the impetus for developing the successful local-tailored models and experiences on malaria control and elimination in Tanzania while providing the platform for expanding the gained experiences and successful model of aid for Africa. With the demonstrated effectiveness in both areas of moderate and high transmission, it is expected that the the strategies and approaches used in this project could be potential for scaling up to other areas of Tanzania and other African countries, to finally accelerate the malaria control and elimination progress in Africa.

In conclusion, this pilot project will not only explore the feasibility of transferring Chinese experiences on malaria control and elimination into African countries, but also provide a model of China-Africa health cooperation through the pilot project led by the tripartite collaboration mechism, technical supports aiming to reduce the disease burden for local population with cost-effective ways.

## Additional file

Additional file 1: Multilingual abstracts in the six official working languages of the United Nations. (PDF 827 kb)
