# Supplementary material for: Application of community-based and integrated strategy to reduce malaria disease burden in southern Tanzania: the study protocol of China-UK-Tanzania pilot project on malaria control
Source: Infect Dis Poverty. 2019 Jan 8;8:4. doi: 10.1186/s40249-018-0507-3 (PMC6334450; doi:10.1186/s40249-018-0507-3)
Supplement: Supplementary file 1 — Multilingual abstracts in the six official working languages of the United Nations. (PDF 827 kb) [file 40249_2018_507_MOESM1_ESM.pdf]

Translation of the abstract into the six official working languages of the United Nations

تطبيق الإستراتيجية المجتمعية والمتكاملة للحد من عبء مرض الملاريا في جنوب تنزانيا: بروتوكول الدراسة للمشروع التجريبي بين الصين والمملكة المتحدة وتنزانيا بشأن مكافحة الملاريا

دوكوان وانغ, بروسبر شاكي، يريمين مالشا، تيجمينو جافانا، ميهايو غابرييل مايكل ، رشيد الخطبو ، يونيو فنغ ، تشنغ-بن تشو ، كانغ مينغ لين ، شانغ شيا ، وهو يان ، ديز ايشنجوما، سوزان روميشا ، سجبرد مكدي، ريناتا مانديكي ، فرانك شاكي ، تشارلز ديسمازي ، سليم عبدالله هونوراتي ماسانجا ، نينغ شياو ، شياو-نونغ تشو

الملخص
خلفية: حققت التجارب الناجحة التي تراكمت في العقود الستة الماضية نجاحا ملحوظا في مكافحة الملاريا والقضاء عليها في الصين ، والتي يمكن مشاركتها مع البلدان الأخرى التي تتوطن فيها الملاريا بما فيها تنزانيا والتي تعاني من عبء كبير من الملاريا. وتعتبر استراتيجية الصين بالمراقبة والاستجابة1-3-7 في الصين للقضاء على الملاريا ، على وجه الخصوص ، أهم التجارب المنتقاة من العديدمن الجهود للعديد من السنوات والابتكارات الرئيسية للقضاء على الملاريا في الصين.
المناهج تم تنفيذ المشروع التجريبي بين الصين والمملكة المتحدة وتنزانيا لمكافحة الملاريا من أبريل 2015 إلى يونيو 2018 ، وكانت عبارة عن دراسة متداخله بين مجتموعتين تتلقي التدخلات المقترحة واثنين من المجتمعات المماثلة التي تعد مواقع تحكم. منظمة الصحة العالمية "تختبر، وتعالج، وتتبيع (مبادرة T3-WHO) ، والتي تدعو كل حالة مشتبه بها لتلقي اختبار تشخيصي ، وكل حالة مؤكدة لعلاجها ، ولتتبع المرضتم دمجها مع الخبرات الصينية في مكافحة الملاريا والقضاء عليها لاستكشاف نموذج مناسب مصمم خصيصًا للظروف المحلية. وبهدف تطبيق استراتيجية الترصد والاستجابة في الصين 1-3-7 لدمج مبادرة T3-WHO والموارد المحلية التي تهدف إلى تقليل عبء الملاريا من حيث خفض معدل الاصابة والوفيات بنسبة 30٪ في مجتمعات التدخل بالمقارنة مع المسح الأساسي الذي أجريت في بداية المشروع.
نقاش: كان المشروع التجريبي بين الصين والمملكة المتحدة وتنزانيا بشأن مكافحة الملاريا هو أول مرة تقوم فيها الصين ببدء مشروع تجريبي لمكافحة الملاريا في قارة أفريقيا، واختبار جدوى التجارب الصينية هناك على شكل تعاون بين الصين وأفريقيا ، *ومن المتوقع أن تكون الاستراتيجيات المستخدمة في هذا المشروع قابلة للتوسع في مناطق أخرى من تنزانيا وغيرها من البلدان الأفريقية ، وفي نهاية المطاف إلى تسريع التقدم في مكافحة الملاريا والتخلص منها في أفريقيا .*

Translated from English version into Arabic by Yasser Atlem, proofread by Aya AlAjjan, through

TRANSLATORS
WITHOUT BORDERS

应用基于社区的综合防治策略降低坦桑尼亚南部地区疟疾疾病负担：中英坦疟疾防治试点项目研究方案

Duoquan Wang, Prosper Chaki, Yeromin Mlacha，Tegemeo Gavana, Mihayo Gabriel Michael, Rashid Khatibu, Jun Feng, Zhengbin Zhou, Kangming Lin, Shang Xia, He Yan, Deus Ishengoma, Susan Rumisha, Sigbert Mkude, Renata Mandike, Frank Chacky, Charles Dismasi, Salim Abdulla , Honorati Masanja, Ning Xiao, Xiao-Nong Zhou

摘要

引言：过去六十年里，中国在疟疾控制和消除方面取得了显著成就，积累了大量的成功经验，值得与世界其他疟疾流行国家分享，其中包括疟疾负担较重的坦桑尼亚。中国用于消除疟疾的“1-3-7”监测和响应策略，是从中国消除疟疾的多年努力和关键创新措施中提炼出的最重要经验。

方法：中英坦疟疾控制试点项目是一项干预性研究，于 2015 年 4 月至 2018 年 6 月在试点地区两个干预社区和两个平行对照社区实施。项目通过使用世界卫生组织“诊断，治疗，跟踪”策略（即对每个疑似病例进行诊断，每个确诊病例进行治疗，以及对治疗后的病例进行跟踪）和中国疟疾控制和消除经验，以探索适合当地环境的适宜工作模式。本研究旨在应用世界卫生组织“诊断，治疗，跟踪”策略，结合中国的“1-3-7”监测响应模式，并充分利用当地现有资源，

减轻干预社区疟疾负担，使干预社区的疟疾发病率和死亡率较项目开始时的基线调查数据降低30%。
讨论：中英坦疟疾控制试点项目是中国首次启动的非洲大陆疟疾控制试点项目，通过中非合作测试中国经验的可行性。该项目所采用的战略有可能扩大到坦桑尼亚其他地区乃至其他非洲国家，并最终加速非洲疟疾控制和消除进程。

Translated from English version into Chinese by Duoquan Wang

Application d'une stratégie communautaire et intégrée pour réduire la charge de morbidité due au paludisme dans le sud de la Tanzanie :*le protocole d'étude du projet pilote Chine-Royaume-Uni-Tanzanie pour la lutte contre le paludisme*

Duoquan Wang, Prosper Chaki, Yeromin Mlacha, Tegemeo Gavana, Mihayo Gabriel Michael, Rashid Khatibu, Jun Feng, Zheng-Bin Zhou, Kang-Ming Lin, Shang Xia, He Yan, Deus Ishengoma, Susan Rumisha, Sigbert Mkude, Renata Mandike, Frank Chacky, Charles Dismasi, Salim Abdulla, Honorati Masanja, Ning Xiao, Xiao-Nong Zhou

Résumé
Contexte: Les expériences réussies accumulées au cours des six dernières décennies ont permis d'obtenir des résultats remarquables en matière de contrôle et d'élimination du paludisme en Chine, qui pourraient être partagés avec d'autres pays d'endémie palustre, dont la Tanzanie durement touchée par ce virus. En particulier, la stratégie de surveillance et de réponse 1-3-7 de la Chine pour l'élimination du paludisme est l'expérience la plus importante et la plus raffinée des efforts de nombreuses années et des principales mesures d'innovation pour l'élimination du paludisme en Chine.
Méthodes: Le projet pilote Chine-Royaume-Uni-Tanzanie sur la lutte contre le paludisme a été mis en œuvre d'avril 2015 à juin 2018, qui était une étude d'intervention avec deux communautés d'intervention recevant les interventions proposées et deux communautés comparables servant de sites témoins. L' Organisation mondiale de la santé "Tester, Traiter, Suivre"（Initiative OMS-T3）, qui demande que chaque cas suspect reçoive un test de diagnostic, que chaque cas confirmé soit traité et que la maladie soit dépistée, a été intégré aux expériences chinoises en matière de contrôle et d'élimination du paludisme afin d'explorer un modèle adapté aux contextes locaux. L'application de la stratégie chinoise de surveillance et et de réponse 1-3-7 intégrée à l'initiative OMS-T3 et aux ressources locales visait à réduire le fardeau du paludisme en termes de réduction de la morbidité et de la mortalité de 30% dans les communautés d'intervention par rapport à l'enquête initiale menée au début du projet.
Discussion: Le projet pilote Chine-Royaume-Uni-Tanzanie pour la lutte contre le paludisme a été la première fois que la Chine a lancé un projet pilote pour la lutte contre le paludisme en Afrique continentale, testant la faisabilité des expériences chinoises dans ce pays sous la forme d'une collaboration sino-africaine, qui devrait permettre d'étendre les stratégies utilisées dans le cadre de ce projet à d'autres régions de la Tanzanie et d'autres pays africains, afin d'accélérer enfin les progrès de la lutte contre le paludisme et de son élimination en Afrique.

Translated from English version into French by Gabriela Kouahla, proofread by Kenza Vionnet, through

TRANSLATORS
WITHOUT BORDERS

Применение общинной и комплексной стратегии по сокращению бремени заболеваемости малярией в Южной Танзании: *протокол исследования пилотного проекта Китай-Великобритания-Танзания по борьбе с малярией*

Дуокван Ван, Проспер Чаки, Еромин Млача, Тегемео Гавана, Михаи Габриэль Майкл, Рашид Хатибу, Джун Фэн, Чжэн-Бин Чжоу, Кан-Минь Линь, Шан Ся, Он Янь, Деус Ишенгома, Сьюзан Румиша, Сигберт Мкуд, Рената Мандике, Фрэнк Чакки, Чарльз Дисмаси, Салим Абдулла, Гонорати Масанья, Нин Сяо, Сяо-Нонг Чжоу

Аннотация
Фон: Успешный опыт, накопленный за последние шесть десятилетий, позволил добиться значительных успехов в борьбе с малярией и ее ликвидации в Китае, которые можно распространить на другие страны, эндемичные по малярии, включая Танзанию, где бремя малярии довольно высокое. В частности, китайская стратегия "1-3-7" по наблюдению и реагированию с целью ликвидации малярии - это важнейший усовершенствованный опыт многолетних усилий и ключевых инновационных мер по ликвидации малярии в Китае.
Методы: Пилотный проект «Китай-Великобритания-Танзания» по борьбе с малярией разрабатывался с апреля 2015 года по июнь 2018 года; это было интервенционное исследование, в котором участвовали два интервенционных сообщества, в которых проводились предлагаемые вмешательства, и два сообщества сравнения, выступающих в качестве контроля. Инициатива Всемирной организации здравоохранения «Test, Treat, Track» (Тестирование, Лечение, Отслеживание) (ВОЗ-Т3), которая призывает в каждом подозрительном случае проводить диагностический тест, в каждом подтвержденном случае проводить лечение и отслеживать каждый случай заболевания, была добавлена к китайскому опыту борьбы с малярией и ее ликвидации с целью исследования надлежащей модели, адаптированной к местным условиям. Применение стратегии наблюдения и реагирования в Китае 1-3-7, вкупе с инициативой ВОЗ-Т3 и местными ресурсами, с целью снижения бремени малярии с точки зрения снижения заболеваемости и смертности на 30% в интервенционных сообществах, по сравнению с базовым исследованием, проведенным в начале проекта,
Обсуждение: Пилотный проект по борьбе с малярией «Китай-Великобритания-Танзания» стал для Китая первым пилотным проектом по борьбе с малярией в континентальной Африке; в нем проверяется применимость китайского опыта в форме китайско-африканского сотрудничества, которое предполагает, что стратегии, используемые в этом проекте, могут быть потенциально распространены на другие районы Танзании и прочие африканские страны с целью в конечном итоге ускорить прогресс в борьбе с малярией и ее ликвидации в Африке.

Translated from English version into Russian by Anfeta, proofread by Natalia Potashnik, through

La aplicación de estrategia basada en la comunidad e integrada con el fin de reducir la incidencia de la enfermedad de la malaria en el sur de Tanzania: *el protocolo de estudio del Proyecto piloto China-Reino Unido-Tanzania sobre el control de la malaria*

Duoquan Wang, Prosper Chaki, Yeromin Mlacha, Tegemeo Gavana, Mihayo Gabriel Michael, Rashid Khatibu, Jun Feng, Zheng-Bin Zhou, Kang-Ming Lin, Shang Xia, He Yan, Deus Ishengoma, Susan Rumisha, Sigbert Mkude, Renata Mandike, Frank Chacky, Charles Dismasi, Salim Abdulla , Honorati Masanja, Ning Xiao, Xiao-Nong Zhou

Resumen
Antecedentes: Las experiencias de éxito de las últimas seis décadas han obtenido logros excepcionales en el control y eliminación de la malaria en China. Dichas experiencias podrían compartirse con otros países donde la malaria es endémica, incluida Tanzania, que tiene una alta incidencia de malaria. Especialmente, la estrategia de vigilancia y respuesta 1-3-7 de China para la eliminación de la malaria constituye la experiencia depurada más importante derivada de los esfuerzos de muchos años y las medidas de innovación clave para la eliminación de la malaria en China.
Metodología: El proyecto piloto China-Reino Unido-Tanzania sobre el control de la malaria se implementó desde abril de 2015 hasta junio de 2018. Era un estudio de intervención con dos comunidades de intervención que recibieron las intervenciones propuestas y dos comunidades

comparables que servían como sitios de control. La iniciativa de la Organización Mundial de la Salud, "Test. Treat. Track” (OMS-T3), que exige que cada caso sospechoso reciba una prueba de diagnóstico, que se asegure el tratamiento de cada caso confirmado y que se haga un seguimiento de la enfermedad, se integró con las experiencias chinas sobre el control de la malaria y su eliminación para estudiar un modelo adecuado adaptado a la configuración local. Con la aplicación de la estrategia de vigilancia y respuesta 1-3-7 de China de forma integrada con la iniciativa OMS-T3 y los recursos locales se pretendía reducir la incidencia de la malaria disminuyendo la morbilidad y la mortalidad en un 30 % en las comunidades de intervención en comparación con la encuesta de referencia que se realizó al inicio del proyecto..

Discusión: El proyecto piloto China-Reino Unido-Tanzania sobre el control de la malaria fue la primera vez que China comenzó un proyecto piloto sobre el control de la malaria en África continental, en el que se probaba la viabilidad de las experiencias chinas allí en forma de colaboración entre China y África. Se espera que las estrategias empleadas en este proyecto puedan servir para la ampliación a otras zonas de Tanzania y en otros países africanos a fin de finalmente acelerar el control de la malaria y el progreso de su eliminación en África.

Translated from English version into Spanish by Jennifer M Lang, proofread by Mar Garcimartín, through

TRANSLATORS
WITHOUT BORDERS
